# Methyl-CpG binding domain protein 2 (Mbd2) drives breast cancer progression through the modulation of epithelial-to-mesenchymal transition

**DOI:** 10.1038/s12276-024-01205-2

**Published:** 2024-04-01

**Authors:** Niaz Mahmood, Ani Arakelian, Moshe Szyf, Shafaat A. Rabbani

**Affiliations:** 1 Department of Medicine, McGill University, Montréal, QC H4A3J1 Canada; 2 Department of Biochemistry, McGill University, Montréal, QC H3A1A3 Canada; 3 Department of Pharmacology and Therapeutics, McGill University, Montréal, QC H3G1Y6 Canada

**Keywords:** Breast cancer, Transcriptomics

## Abstract

Methyl-CpG-binding domain protein 2 (Mbd2), a reader of DNA methylation, has been implicated in different types of malignancies, including breast cancer. However, the exact role of Mbd2 in various stages of breast cancer growth and progression in vivo has not been determined. To test whether Mbd2 plays a causal role in mammary tumor growth and metastasis, we performed genetic knockout (KO) of *Mbd2* in MMTV-PyMT transgenic mice and compared mammary tumor progression kinetics between the wild-type (PyMT-*Mbd2*^*+/+*^) and KO (PyMT-*Mbd2*^−/−^) groups. Our results demonstrated that deletion of *Mbd2* in PyMT mice impedes primary tumor growth and lung metastasis at the experimental endpoint (postnatal week 20). Transcriptomic and proteomic analyses of primary tumors revealed that Mbd2 deletion abrogates the expression of several key determinants involved in epithelial-to-mesenchymal transition, such as neural cadherin (N-cadherin) and osteopontin. Importantly, loss of the *Mbd2* gene impairs the activation of the PI3K/AKT pathway, which is required for PyMT-mediated oncogenic transformation, growth, and survival of breast tumor cells. Taken together, the results of this study provide a rationale for further development of epigenetic therapies targeting Mbd2 to inhibit the progression of breast cancer.

## Targeting Mbd2 for epigenetic therapy in breast cancer

DNA methylation, a process that regulates how genes work, is often disturbed in cancer. This research looked at the role of Methyl-CpG-Binding Domain Protein 2 (MBD2), a protein that interprets these methylation signals, in breast cancer. It was found that MBD2 is overly active in several kinds of cancer, including breast cancer. In an experiment with mice, it was found that removing MBD2 slowed down the formation of tumors and reduced the spread of cancer. MBD2 was found to control several genes involved in how cancer progresses and how the immune system responds. The research suggests that focusing on MBD2 could be a possible treatment approach for breast cancer.

This summary was initially drafted using artificial intelligence, then revised and fact-checked by the author.

## Introduction

DNA methylation is an epigenetic modification that defines cell identity by modulating chromatin architecture and gene expression^1^. This chemically reversible process is mediated by a family of “writer” enzymes known as DNA methyltransferases (DNMTs) that catalyze the addition of methyl (-CH_3_) moieties to the fifth position of cytosine residues in most mammalian genomes^2^. A family of evolutionarily conserved epigenetic “reader” proteins known as methyl-binding proteins (MBPs) then recognize, interpret, and relay the information from these methylation marks for different gene regulatory functionalities^3^.

Dysregulated DNA methylation is associated with various pathological conditions, including cancer^4^. As such, nonmutational epigenetic reprogramming is recognized as a paradigmatic hallmark of human cancer^5^ and an attractive anticancer therapeutic target. The currently approved epigenetic drugs targeting DNMTs, namely, azacitidine and decitabine, have shown robust clinical utility against several hematological malignancies^6^. However, these drugs are highly toxic, have poor bioavailability, and display only a modest anticancer effect in solid tumors^7^. Moreover, these inhibitors cause global demethylation that relieves the transcriptional repression of critical tumor suppressors^8^ but also causes undesired transcriptional activation of several known prometastatic genes^9^. This indicates the need to explore novel strategies to selectively target other epigenetic modulators, such as MBPs, as alternative approaches to reversing DNA methylation-mediated epigenetic abnormalities in cancer.

Among the MBPs, methyl-CpG-binding domain protein 2 (Mbd2) is positioned as a suitable anticancer drug target since its expression is frequently upregulated in several human malignancies^3^. Compared to other MBPs, Mbd2 shows a relatively greater affinity for methylated DNA in the promoter regions of several tumor suppressor genes, causing their transcriptional repression^10^. Mice with genetic knockout (KO) of the *Mbd2* gene produced viable offspring, suggesting that the gene is not required to maintain standard physiological functions^11^ and thus could be used for targeted epigenetic therapies. Indeed, genetic KO of *Mbd2* protects mice from developing intestinal^12^ and lymphoid malignancies^13^. Targeting the human *MBD2* gene with an antisense oligonucleotide inhibited the growth of lung and colorectal cancer xenografts in vivo^14^. More recently, hypoxia-induced expression of the canonical MBD2a isoform was shown to promote epithelial-to-mesenchymal transition (EMT) through activation of the Wnt/β-catenin signaling pathway receptor frizzled class receptor 1 (FZD1)^15^.

The role of Mbd2 in breast cancer progression has thus far been studied through gene knockdown in vitro and subsequent implantation of the generated cells into animals to establish xenograft models, in which Mbd2 depletion has shown potent anticancer effects through the inhibition and hypermethylation of prometastatic genes^16,17^. However, these model animals lack functional immune systems, and the exact role of Mbd2 in the highly complex multistep progression of human breast tumors thus cannot be fully replicated. Moreover, in previous studies, complete KO of the gene encoding Mbd2 was not performed^16,17^. In a KO model, the target gene is completely deleted or inactivated in the organism’s genome, allowing a more direct assessment of the gene’s function and impact on cancer development. There are several other advantages of using transgenic KO models to study the role of Mbd2 in breast tumor progression. These models allow the investigation of the systemic effects of deletion of the selected gene. Global loss of the gene can reveal its potential roles in other tissues or organs that may contribute to cancer development or progression^18^. In xenograft models, gene deletion is limited to the transplanted cancer cells and may not fully reflect the systemic influence of a gene. The transgenic model allows longitudinal studies over the entire lifespan of the organism, enabling the investigation of the gene’s role in cancer initiation, progression, and metastasis. In xenograft models, the study duration is typically limited due to the short lifespan of the mice and the growth kinetics of the transplanted tumors. To determine the role of Mbd2 in a mouse model with a relatively faithful representation of breast tumor progression, we used a transgenic MMTV-PyMT (mouse mammary tumor virus-polyoma middle tumor-antigen) model, in which the spontaneous and pregnancy-independent expression of the PyMT oncoprotein results in the synchronous appearance of multifocal breast tumors that metastasize predominantly to the lung^19,20^. Although the PyMT oncoprotein is not expressed in human breast tumors, and other triggers initiate breast cancer, the stepwise progression of the mammary tumors in these mice from a benign premalignant stage to a highly malignant invasive stage and the activation of downstream molecular signaling pathways resemble those observed during breast cancer progression in humans^21^. These properties position the PyMT model as a system that can be used to assess the oncogenic function of a particular gene in the evolution of the malignant transformation of breast cells in an organism-wide manner^22^.

Herein, using a molecular genetics approach in transgenic MMTV-PyMT mice, we demonstrated that *Mbd2* deletion significantly decreases mammary tumor growth and metastasis.

## Materials and methods

### Mouse strains and genotyping

The embryo straws for *Mbd2* heterozygous KO (*Mbd2*^+/−^) mice on the C57BL/6 background were kindly provided by Dr. Brian Hendrich (Department of Biochemistry, University of Cambridge). The embryos were first recovered from the straws at the Transgenic Core Facility, Rosalind and Morris Goodman Cancer Research Centre, McGill University. Then, they were implanted into two female foster mice to generate heterozygous KO (*Mbd2*^+/−^) mice. Afterward, the mice were bred and maintained at the Animal Resource Division of the Research Institute of the McGill University Health Center. We purchased the breeder pair, i.e., a male heterozygous MMTV-PyMT mouse (on the C57BL/6 background) carrying a single copy of the PyMT transgene and a noncarrier wild-type C57BL/6 female mouse, from The Jackson Laboratory (Bar Harbor, ME, USA) and bred them to obtain male and female MMTV-PyMT mice (PyMT) for the study. Male PyMT mice were crossed with female heterozygous *Mbd2*^+/−^ mice to obtain PyMT-*Mbd2*^+/−^ (heterozygous KO of *Mbd2*) littermates. Next, male PyMT-*Mbd2*^*+/−*^ and female *Mbd2*^*+/−*^ mice were crossed to obtain female PyMT-*Mbd2*^*+/+*^, PyMT-*Mbd2*^*+/−*^, and PyMT-*Mbd2*^−/−^ (homozygous KO of *Mbd2*) mice. All mice were heterozygous for the PyMT transgene. The PyMT-*Mbd2*^+/+^ and PyMT-*Mbd2*^−/−^ mice were used for experimental purposes.

For genotyping, DNA was extracted from the tails of mice aged less than 3 weeks using the AccuStart II PCR Genotyping Kit (Quantabio, MA, USA; Cat # 95135-100). Next, polymerase chain reaction (PCR) was performed using primers to detect specific alleles. The PyMT allele was detected using the following primers (5’ → 3’): GGAAGCAAGTACTTCACAAGGG (forward) and GGAAGTCACTAGGAGCAGGG (reverse). The *Mbd2*-KO and corresponding WT mice were genotyped using a combination of three primers (5’ → 3’): TTGTGAGCTGTTGGCATTGT, GTCAACAGCATTTCCCAGGT, and TGTCCTCCAGTCTCCTCCAC. When the PCR-amplified products were run on an agarose gel, the wild-type mice (*Mbd2*^*+/+*^) were identified by the presence of a single band with a size of 377 bp; the homozygous KO mice (*Mbd2*^*−/−*^), by a single 250 bp product; and the heterozygous KO mice (*Mbd2*^*+/−*^), by bands for both the 377 and 250 bp products.

Once the mice started to develop palpable mammary tumors spontaneously, the size of the tumors was measured at weekly intervals using a Vernier caliper, and the tumor volume was calculated by the following equation: V = (length × Width2)/2, as we described previously in ref. ^23^.

### Immunoblotting

Immunoblotting was performed on snap-frozen mouse primary tumor tissues obtained by mechanically crushing the tumors under cryogenic conditions (in liquid nitrogen) using a mortar and pestle. We used radioimmunoprecipitation assay (RIPA) buffer (150 mM NaCl, 1% NP-40, 0.5% sodium deoxycholate, 0.1% sodium dodecyl sulfate (SDS), and 50 mM Tris [pH 7.4]) supplemented with a mixture of appropriate protease and phosphatase inhibitors to prepare the lysates. Following the quantification of protein concentrations using the Bradford assay, 15 µg of protein from each sample was resuspended in Laemmli buffer, boiled at 95 °C for 5 min, electrophoresed on 8 to 15% sodium dodecyl sulfate‒polyacrylamide gels that were prepared in-house, and transferred to a polyvinylidene difluoride (PVDF) membrane (Bio-Rad, Hercules, CA, USA; Cat# 1620177) at 4 °C. The membrane was first immersed in 5% skim milk in Tris-buffered saline supplemented with 0.1% Tween 20 detergent (TBST) to block nonspecific antibody binding and then incubated with appropriate primary antibodies obtained from different commercial vendors. Afterward, the membrane was washed with TBST and incubated with appropriate horseradish peroxidase-conjugated secondary antibodies (all secondary antibodies were obtained from Bio-Rad) for 1 h. The membrane was subsequently washed with TBST three times, and an enhanced chemiluminescence detection kit (GE Healthcare Life Sciences, Amersham, UK, Cat# RPN2232) was used for visualization of protein bands via a ChemiDoc MP Imaging System (Bio-Rad Laboratories, Inc., Hercules, CA, USA). The antibodies used in this study were as follows: anti-MBD2 (EpiGentek and Abcam), anti-p-c-Src (Santa Cruz Biotechnology), c-Src (Santa Cruz Biotechnology), anti-p-PI3K (Cell Signaling Technology), anti-PI3K (Cell Signaling Technology), anti-p-AKT (Cell Signaling Technology), anti-AKT (Cell Signaling Technology), anti-OPN (Assay designs), anti-N-Cadherin (Novus Biologicals), anti-c-Myc (Santa Cruz Biotechnology), anti-β-actin (Sigma‒Aldrich), and anti-GAPDH (EMD Millipore).

### RNA extraction and quantitative polymerase chain reaction (qPCR)

Total RNA was extracted using an AllPrep DNA/RNA Mini Kit (QIAGEN, Hilden, Germany; Cat# 80204). The RNA concentration was measured using a BioDrop spectrophotometer (Montreal Biotech, Inc., QC, Canada), and 2 µg of total RNA from each sample was subjected to reverse transcription‒polymerase chain reaction (RT‒PCR) with random hexamer primers (Invitrogen, Waltham, MA, USA; Cat# 58875). SYBR^®^ Green (Applied Biosystems, Cat#A25742)-based quantitative PCR (qPCR) analysis was performed using an ABI StepOnePlus™ (Applied Biosystems) instrument. The qPCR primers used in this study are listed in Supplementary Table 1a, b. All primers were obtained from Integrated DNA Technologies. Differences in gene expression between the control and KO groups were determined using the 2^-ΔΔCt^ method described by Livak and Schmittgen^24^.

### Chromatin immunoprecipitation followed by sequencing (ChIP-Seq)

At the experimental endpoint of the study, primary breast tumors from wild-type and *Mbd2 KO* PyMT mice were rapidly collected, flash-frozen and stored at −80 °C until further experiments were performed. For ChIP-Seq, tumor tissue from three individual wild-type PyMT mice was used (*n* = 3), and ChIP-Seq data from an *Mbd2* KO PyMT tumor was used as a control for signal normalization during bioinformatic analyses. MBD2-bound DNA was isolated using the EpiQuik Tissue Methyl-CpG Binding Domain Protein 2 ChIP Kit (EpiGentek, NY, USA; Cat# P-2018-48) according to the protocol provided by the manufacturer. The mouse anti-MBD2 antibody used for the ChIP experiment was included in the kit. The DNA obtained from the different samples was subsequently sent to the Génome Québec sequencing facility; the DNA quality check (with an Agilent 2100 Bioanalyzer), library preparation (with an NEB Ultra II Kit), and subsequent sequencing (with the NovaSeq 6000 System) steps were performed at McGill University and the Génome Québec Innovation Centre, and the data analyses were performed at the Bioinformatics Core Facility, Institut de Recherches Cliniques de Montréal (IRCM), Montreal. The quality of the sequencing reads was assessed via FastQC (http://www.bioinformatics.babraham.ac.uk/projects/fastqc/). The reads were subsequently aligned to the mouse genome (mm10) using Bowtie 2^25^. MACS^26^ was used to identify the MBD2-enriched regions pulled down by the antibody. The Chip-Seq reads obtained from an *Mbd2-*KO PyMT tumor were used to eliminate nonspecific peaks. Only the narrow peaks with a cutoff *P* value of less than 0.05 identified by MACS were selected as the Mbd2-occupied regions. To determine the de novo and previously annotated transcription factor motifs within/proximal to the Mbd2-enriched regions, HOMER^27^ was used. ConsensusPathDB^28^ was used for pathway analyses. For ChIP‒qPCR, an IgG isotype control was used for normalization of Mbd2 enrichment near the regulatory region of *Foxp3*. The ChIP primers used are listed in Supplementary Table 1c.

### RNA-Seq and analysis pipeline

RNA was extracted from mammary tumors of PyMT-*Mbd2*^*+/+*^ and PyMT-*Mbd2*^*−/−*^ mice and subjected to RNA sequencing (*n* = 3 mice/group). First, the integrity of the RNA was checked and confirmed with an Agilent 2100 Bioanalyzer, and ribosomal RNA was subsequently removed from the samples with a Ribo-Zero Kit (Illumina, San Diego, CA, United States). The sequencing library was prepared following the standard protocol for the TruSeq Stranded Total RNA Sample Prep Kit (Illumina), and paired-end [2 × 150 bp] sequencing was performed on the NovaSeq 6000 sequencing system (Illumina) at LC Sciences (Houston, USA). Once the sequencing run was completed, the adaptor sequences and low-quality and undetermined bases were removed, and the quality of the sequence reads was verified by FastQC (http://www.bioinformatics.babraham.ac.uk/projects/fastqc/). The demultiplexed reads were subsequently mapped to the reference genome of *Mus musculus* (version v90) using the Bowtie2^25^ and HISAT2^29^ aligners. The assembly of the mapped sequencing reads and differential expression of the transcripts were estimated using StringTie^30^ and edgeR^31^, respectively. Known long noncoding RNAs (lncRNAs) were identified based on sequence similarities. To identify novel long noncoding RNAs (lncRNAs), we first filtered out the transcripts that overlapped with the known mRNAs or the known lncRNAs and transcripts shorter than 200 bp. The Coding Potential Calculator (CPC)^32^ and Coding-Non-Coding-Index (CNCI)^33^ tools were subsequently used to predict the transcripts with coding potential. The transcripts with a CPC score < −1 and a CNCI score < 0 were filtered out, and the remaining transcripts were considered novel lncRNAs. The lists of known and novel lncRNAs were subsequently combined and used for downstream analyses. For both mRNAs and lncRNAs, the following two criteria were used to identify differentially expressed transcripts (1) log_2_ (fold change) greater than 1 or log_2_ (fold change) less than −1 and (2) *P* value < 0.05 (parametric F test comparing nested linear models). Pathway analyses were performed by using ConsensusPathDB^28^.

### Proteomic analysis of the tumor samples

Protein lysates obtained from homogenized mammary tumors of PyMT-*Mbd2*^*+/+*^ and PyMT-*Mbd2*^*−/−*^ mice were subjected to proteomic profiling using ultra-high-performance liquid chromatography–tandem mass spectrometry (UHPLC/MS-MS) at the RI-MUHC proteomics core (*n* = 3 samples/group). For the identification of peptides and proteins, Scaffold software (version 4.9; Proteome Software, Inc., Portland, OR, USA) was used^34^. The cutoff probabilities for identification of peptides and proteins were set at minimums of 90% and 95%, respectively (a minimum of two peptides). A *P* value cutoff of less than or equal to 0.05 was considered to indicate statistical significance for identifying proteins with differential abundances in the two groups.

### Immunohistochemistry

Formalin-fixed mammary tumor tissues were stained with a monoclonal antibody against Ki67 (Dako, Cat# M7240), and the number of Ki67-positive cells was determined from photomicrographs of five randomly selected fields for each sample with the ‘ImmunoRatio’ plugin^35^. To identify Foxp3+ T-regs, formalin-fixed mammary tumor tissues were double stained with antibodies against CD3 (Dako, Cat# A0452) and Foxp3 (Novus Biologicals, Cat# NB100-39002) via the histopathology platform of RI-MUHC, Montréal, QC, Canada. For each sample, seven randomly selected fields were photographed, and the tumor cells exhibiting positive staining for CD3 and Foxp3 were manually counted.

### Cell culture, siRNA transfection and invasion assay

Human MDA-MB-231 cells were initially obtained from the American Type Culture Collection (ATCC® HTB- 26™; Manassas, Virginia). The mouse PyMT-R221A and E0771 breast cancer cell lines were kindly provided by Dr. Conor C. Lynch (H. Lee Moffitt Cancer Center and Research Institute, Tampa, FL, USA) and Dr. Peter Siegel (McGill University, Montreal, Québec, Canada), respectively. For all three cell lines used in this study [human MDA-MB-231, mouse PyMT-R221A and E0771 cells], Dulbecco’s modified Eagle’s medium (DMEM) (Wisent, Saint-Jean-Baptiste QC, Canada; Cat# 319-015-CL) supplemented with 10% fetal bovine serum (FBS) (Wisent; Cat# 085450) and antibiotic-antimycotic solution (Wisent; Cat#450-115-EL) was used as the growth medium. Before starting the in vitro experiments, the cells were passaged at least twice after initial resuscitation from cryogenic storage. A schematic of the in vitro treatment protocol using mouse breast cancer cell lines is shown in Fig. 6h. Briefly, E0771 and PyMT-R221A cells were plated in 6-well plates and allowed to adhere for 24 h. The next day, the cells were treated with recombinant Transforming growth factor beta (rTGF-β). The following day, small interfering RNA (siRNA) against *Mbd2* (siMbd2) or scrambled siRNA was transfected into the cells using Lipofectamine 2000 (Invitrogen, CA, USA) following the manufacturer’s protocol. Twenty-four hours after transfection, cells treated with the control (scrambled siRNA), rTGF-β (10 ng/mL), siMbd2 (final concentration, 30 nM, Santa Cruz Biotechnology, TX, USA; Cat# sc-35866) or rTGF-β+siMbd2 were harvested using trypsin, and the number of cells was determined. Afterward, the invasion assay was carried out using a two-compartment Boyden chamber (Costar Transwell, Sigma‒Aldrich) following the protocol described previously^36^.

### CRISPR-Cas9-mediated *MBD2* depletion

We first generated an MDA-MB-231 cell line stably expressing the Cas9 protein via lentiviral transduction. For the production of lentiviruses containing the Cas9 plasmid, we cotransfected the lentiCas9-Blast plasmid (Addgene, plasmid #52962) with the psPAX2 (Addgene, plasmid #12260) and pMD2.G (Addgene, plasmid #12259) plasmids into HEK293T cells using Lipofectamine 2000 transfection reagent following the manufacturer’s instructions. After forty-eight hours of transfection, we collected the supernatant, filtered it through a 0.45 μM filter and stored it at −80 °C until further use. One day prior to transduction, 3 × 10^5^ MDA-MB-231 cells were plated in 12-well plates. Polybrene (6 μg/mL) was used for lentiviral transduction. We started the selection of transduced MDA-MB-231 cells using the antibiotic blasticidin (75 μg/mL) two days after transduction. All cells in the control group (without transduction) died three days after blasticidin treatment. We then propagated the Cas9-expressing cells for two more weeks in growth medium containing blasticidin before starting the transfection for CRISPR-mediated depletion of the *MBD2* gene.

For CRISPR-mediated gene ablation, 6 × 10^5^ MDA-MB-231-Cas9 cells were plated in each well of a 6-well plate and transfected with either a scrambled gRNA plasmid [pLenti-gRNA-puro (Addgene, plasmid #180426)] or a gRNA plasmid to deplete *MBD2*. In the MBD2 gRNA plasmid, the scrambled gRNA sequence (GCACTACCAGAGCTAACTCA) in the pLenti-gRNA-puro (Addgene, plasmid #180426) plasmid was used to replace the *MBD2*-targeting sequence (gCCAGGTACCTTGCCAACTG) where ‘g’ indicates a base noncomplementary to the MBD2 target sequence but necessary to initiate the transcription of the gRNA from the U6 promoter. Transfection was performed using Lipofectamine 2000 transfection reagent following the manufacturer’s instructions. Two days after transfection, cells expressing the gRNA plasmids were selected using puromycin (2 μg/mL) for 2-3 days. The surviving cells were passaged several times with growth medium containing puromycin for two additional weeks. Depletion of the *MBD2* gene was confirmed by western blotting. For the rescue experiment, MBD2-depleted MDA-MB-231 cells were transfected with plasmids expressing either the MBD2a [MBD2a pcDNA3.1 (Addgene, plasmid #78141)] or MBD2b [MBD2b pcDNA3.1 (Addgene, plasmid #78142)] protein, the expression of which was confirmed by the presence of the respective bands on a western blot.

### Statistical analyses

The data in the different graphical representations are shown as the mean ± standard error of the mean (SEM) values unless otherwise indicated. Depending on the number of groups analyzed, Student’s *t*-test or ANOVA was used to determine the statistical significance of the differences. A *P*-value less than or equal to 0.05 was considered to indicate statistical significance.

## Results

### Mbd2 expression is upregulated in mammary tumors

**Fig. 1:**
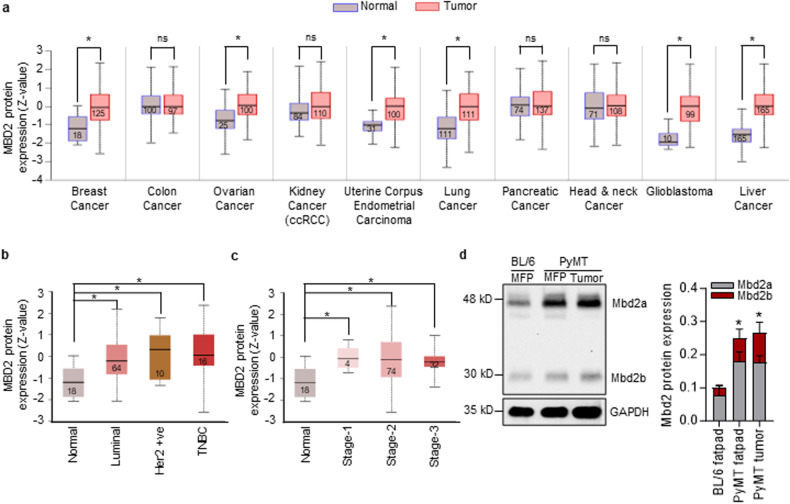
Mbd2 expression is elevated in tumor tissues. **a** Boxplots showing human MBD2 protein levels in different types of cancer tissues and their normal counterparts according to CPTAC datasets. Statistically significant differences are marked by asterisks, and the number of patients in each group is indicated within each box. Higher expression of MBD2 was observed in tissues from patients with different breast cancer subtypes (**b**) and at different stages (**c**) of breast cancer. **d** A representative immunoblot of the mouse Mbd2 protein from lysates obtained from the mammary fat pads (mfp) of 11-week-old female C57BL/6 (Lane 1) and PyMT mice (Lane 2) and mammary tumors from 20-week-old PyMT mice (Lane 3). GAPDH was used as a loading control. The right panel shows normalized densitometric quantification of the total Mbd2 signal (*n* = 3 mice/group). The results are shown as the means ± SEMs. Statistical significance was determined via quantitative analysis of total Mbd2 protein expression (sum of Mbd2a and Mbd2b) using ANOVA followed by Tukey’s *post hoc* test. Statistical significance is indicated by an asterisk.

We first interrogated publicly available proteomics datasets from the Clinical Proteomic Tumor Analysis Consortium (CPTAC) using the UALCAN portal^37^ and found that MBD2 expression was significantly upregulated in tissues of breast cancer and several other common types of cancer compared to the normal counterparts (Fig. 1a). Moreover, the MBD2 protein level was significantly elevated in the well-known molecular subtypes of human breast tumors [luminal, Her2-positive, and triple-negative breast cancer (TNBC)] (Fig. 1b). When patients were stratified according to the stage, the expression of human MBD2 protein significantly elevated in all three stages relative to that in normal healthy controls (Fig. 1c). Moreover, Mbd2 protein expression was markedly increased in mammary fat pads (mfp) and primary tumors obtained from transgenic PyMT mice compared to the fat pads of WT C57BL/6 mice, indicating that Mbd2 upregulation mediated by the *PyMT* gene precedes the appearance of measurable mammary tumors (Fig. 1d).

### Deletion of Mbd2 attenuates mammary tumor progression in a transgenic MMTV-PyMT model

**Fig. 2:**
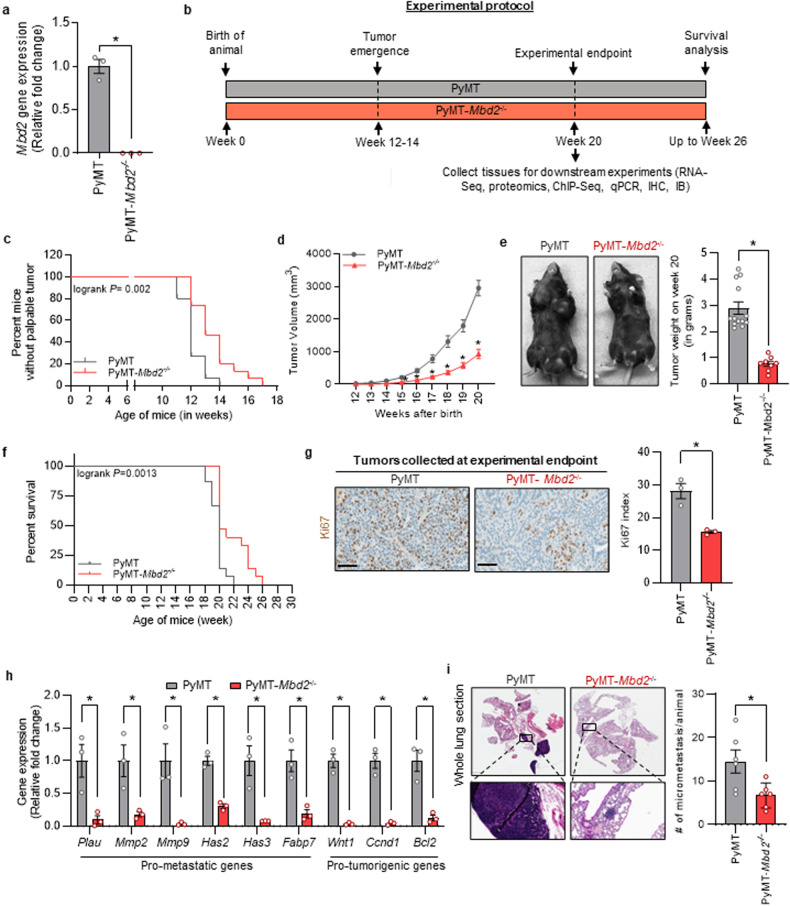
Deletion of *Mbd2* gene affects tumor progression in transgenic PyMT mice. **a** qPCR confirming KO of *Mbd2* gene in PyMT-*Mbd2*^*−/−*^ tumors (*n* = 3/group). **b** Schematic representation of the endpoints of this study. **c** A Kaplan‒Meier curve showing tumor emergence in the PyMT and PyMT-*Mbd2*^*−/−*^ groups. **d** Tumor volumes in wild-type and *Mbd2*-KO PyMT mice were measured at weekly intervals (*n* = 15 mice/group). **e** Tumor weight in each group was measured at the experimental endpoint. At least eight mice in each group were sacrificed at this point, in addition to those bearing tumors that reached a volume requiring humane sacrifice (*n* = 13 in the PyMT group and *n* = 8 in the PyMT-*Mbd2*^*−/−*^ group). The remaining mice were kept for survival analysis, as shown in (**f**). It should be noted that the survival curve contains data for all the mice used for the study (15 mice/group). **g** Immunohistochemical staining of the primary tumors from each group of mice with an antibody against the proliferation marker Ki67 (left panel; scale bar = 60 µm). The percentage of Ki67-positive tumor cells was determined from five high-power fields for each sample and is plotted as a bar graph in the right panel (*n* = 3 mice/group). **h** qPCR analysis of several known cancer-related genes using RNA extracted from primary tumors from wild-type and PyMT-*Mbd2*^*−/−*^ mice (*n* = 3 mice/group). **i** H&E staining of formalin-fixed lung tissues obtained at the experimental endpoint (left panel). The number of micrometastases was determined and plotted as a bar graph (*n* = 6 mice/group). The results are shown as the means ± SEMs. Statistical significance was determined using Student’s *t*-test and is shown by an asterisk.

To understand the role of Mbd2 in mammary tumor growth and metastasis, we first generated PyMT-*Mbd2*^*−/−*^ (homozygous *Mbd2-*KO) mice on a C57BL/6 background using the crossbreeding strategy described in the “Materials and Methods” section and confirmed the deletion of *Mbd2* gene in the primary tumors by qPCR (Fig. 2a). Next, we compared the tumor growth kinetics between the PyMT-*Mbd2*^*−/−*^ group and the control PyMT-*Mbd2*^*+/+*^ group from postnatal week 11 until sacrifice (scheme in Fig. 2b). The Kaplan‒Meier curve in Fig. 2c shows that mice from the PyMT group started to develop palpable tumors at approximately postnatal week 11. By week 14 of age, all mice in the PyMT group had developed primary tumors [time to PyMT tumor incidence: 11-14 weeks]. The initiation of palpable tumors was delayed in the PyMT-*Mbd2*^*−/−*^ group compared to that in the wild-type PyMT-*Mbd2*^*+/+*^ group (log–rank *P* = 0.002), in which tumors started to develop beginning in week 12, and all the KO mice had developed palpable tumors by week 16 of age [time to PyMT-*Mbd2*^*−/−*^ tumor incidence: 12–16 weeks], suggesting the possible involvement of Mbd2 in tumorigenesis in this model. We assessed the primary tumor volumes at weekly intervals from the time that measurable tumors were detected in wild-type PyMT mice to the time of sacrifice (*n* = 15 mice/group). The experimental endpoint was set at postnatal week 20, when most wild-type mice had reached the humane endpoint. In some wild-type mice, the tumor volume reached the human endpoint before week 20, at which point the mice were sacrificed. We found that the tumor growth rate was significantly reduced in the PyMT-*Mbd2*^*−/−*^ group (Fig. 2d), an observation consistent with similar results in intestinal tumorigenesis^12^. At week 20, at least 8 mice per group (*n* = 13 in the PyMT group and n = 8 in the PyMT-*Mbd2*^*−/−*^ group) were sacrificed. We then measured the total weight of the excised tumors of the mice sacrificed before or at postnatal week 20 and found a significant reduction in tumor weight in the *Mbd2*-KO group compared to the wild-type group (Fig. 2e). To test whether Mbd2 plays a role in prolonging the survival of mammary tumor-bearing mice, we kept several mice from each group beyond the experimental endpoint at week 20 (after birth). We found that the tumor volumes in the mice in the homozygous KO group reached the humane endpoint requiring humane sacrifice later than those in the mice in the wild-type group (Fig. 2f). All mice in the PyMT-*Mbd2*^*+/+*^ and PyMT-*Mbd2*^*−/−*^ groups had to be sacrificed by week 22 and week 26, respectively, as the tumor volumes reached the humane endpoint.

We next performed immunohistochemistry on formalin-fixed tumors excised at the experimental endpoint. Consistent with the decrease in tumor volume shown in Fig. 2d, we found significant downregulation of the proliferation marker Ki67 in the tumors from the PyMT-*Mbd2*^*−/−*^ group compared to those from the wild-type group (Fig. 2g). Next, using RNA from flash-frozen tumor tissues collected at the experimental endpoint, we measured the expression of several known cancer-related genes [plasminogen activator urokinase (*Plau*), matrix metallopeptidase 2 (*Mmp2*)*, Mmp9*, hyaluronan synthase 2 (*Has2*)*, Has3*, fatty acid binding protein 7 (*Fabp7*), Wnt family member 1 (*Wnt1*), cyclin D1 (*Ccdn1*), and B-cell lymphoma 2 (*Bcl2*)] that were previously shown to be upregulated either directly through Mbd2 or indirectly through its downstream signaling pathways^17,38^, and we found that their expression was significantly decreased in PyMT-*Mbd2*^*−/−*^ tumors compared to wild-type tumors (Fig. 2h). Since several prometastatic genes (*Plau, Mmp2, Mmp9, Has2*, and *Has3*) were downregulated in *Mbd2*-KO PyMT tumors, we next used formalin-fixed lung tissue sections collected at the experimental endpoint and stained them with hematoxylin and eosin (H&E) to assess breast tumor metastasis to the lung. We found a significant decrease in the number of micrometastases in the lungs harvested from the Mbd2-deleted mice compared to those harvested from the wild-type PyMT mice (Fig. 2i). These results are consistent with those of other studies demonstrating a crucial role for Mbd2 in breast cancer metastasis^39,40^.

### Mbd2 deletion inhibits PyMT-dependent activation of the PI3K/AKT axis

**Fig. 3:**
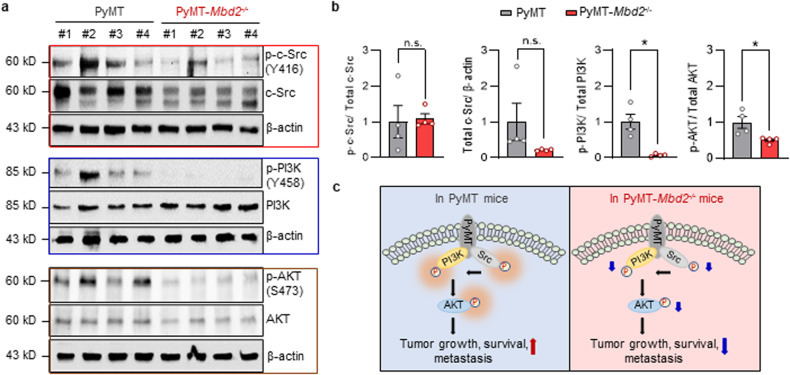
Deletion of Mbd2 attenuates the PyMT-induced oncogenic PI3K/AKT pathway. **a** Immunoblots of whole-tumor lysates obtained from four individual mice/group (left panel). The bands with locations corresponding to the known molecular weight of each protein are shown. β-Actin was used as a loading control. **b** Densitometric quantification of the bands for each protein was performed, and the results are presented as the means ± SEMs (*n* = 4 tumors/group) (right panel). Statistical significance was determined using Student’s *t-*test and is shown by an asterisk. n.s.= not significant. **c** Schematic diagram of PyMT-mediated oncogenic signaling and the downregulation of the corresponding proteins upon *Mbd2* deletion. The left panel shows the phosphorylation-mediated activation of PI3K/AKT signaling in wild-type PyMT tumors, which led to enhanced tumor growth and metastasis. The right panel shows the reduced activation of the PI3K/AKT signaling pathway upon Mbd2 deletion, which is a likely cause of the reduced tumor growth and metastasis observed in the transgenic PyMT-*Mbd2*^*−/−*^ mice.

PyMT is a membrane-associated oncoprotein of viral origin with no intrinsic kinase activity^22^. However, when it interacts with receptor tyrosine kinases (RTKs) [for example, c-Src, the p85 subunit of phosphoinositide 3-kinase (PI3K)], the resultant protein complex gains the constitutive tyrosine kinase activity required for the activation of downstream signaling pathways to promote cellular transformation, growth, and survival^19^. To examine whether Mbd2 deletion directly impairs the downstream effectors of PyMT-mediated oncogenic signaling, we measured the levels of activated c-Src, PI3K, and AKT in protein extracts obtained from wild-type and *Mbd2-*KO PyMT tumors. When the level of Y416-phosphorylated c-Src was normalized to the total c-Src protein level, there was no significant difference in the level of p-c-Src (Y416) between the wild-type and *Mbd2-*KO PyMT tumors. However, there was a substantial decrease in the total c-Src level in *Mbd2-*KO PyMT tumors, which, in turn, caused a net reduction in the p-c-Src (Y416) level (Fig. 3a, b). *Src* gene expression did not differ between wild-type and Mbd2-deleted PyMT tumors (Supplementary Fig. 1), thus eliminating the possibility that *Src* is transcriptionally regulated by the Mbd2 protein. However, Mbd2 may indirectly mediate the regulation of Src protein expression in these tumors at the level of mRNA translation or stability, a possibility that needs to be tested in the future. We found that PI3K activation was significantly impaired in *Mbd2*^*−/−*^ tumors compared with the wild-type tumors, as shown by the significant reduction in PI3K phosphorylation at Y458 in tumors from KO mice (Fig. 3a, b). In addition, significant impairment of the activation of AKT (i.e., phosphorylation at S473), which is downstream of the PI3K signaling pathway, was observed in tumors from the PyMT-*Mbd2*^*−/−*^ group compared to their wild-type counterparts (Fig. 3a). Taken together, these data suggest that deletion of the *Mbd2* gene impairs the ability of the PyMT oncoprotein to activate the oncogenic PI3K/AKT pathway, which is involved in cell growth, survival, and metastasis (Fig. 3c).

### The Mbd2 binding landscape in heterogeneous PyMT tumors

**Fig. 4:**
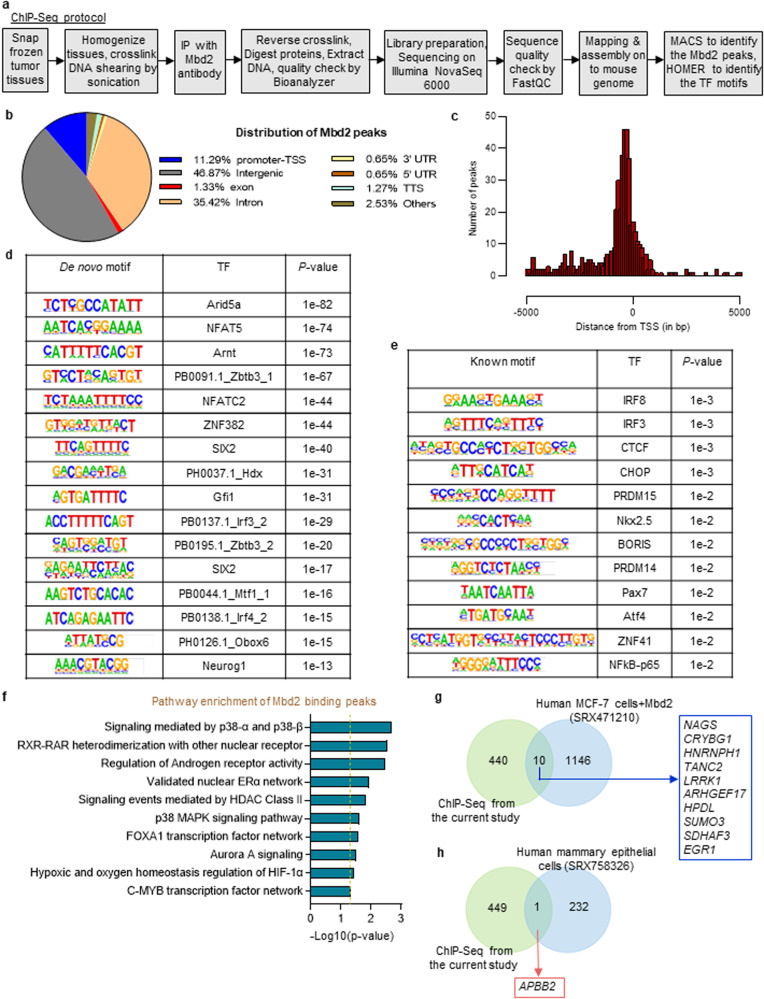
The genomic landscape of Mbd2 binding in PyMT tumors. **a** Schematic of the steps followed for ChIP-Seq. **b** Pie chart showing the genomic distribution of the corresponding targets bound by Mbd2. Here, TSS: transcription start site, UTR: untranslated region, TTS: transcription termination site. **c** Enrichment of Mbd2 binding peaks at genomic positions relative to the TSS (within 5000 bp up- or downstream of the TSS). Logos of HOMER-predicted de novo (**d**) and known (**e**) motifs that are significantly enriched within or near the Mbd2 binding sites. Here, TF represents the associated transcription factor that can potentially recognize the respective motif. **f** Pathway analysis of the annotated genes nearest genes with Mbd2 binding peaks using the ConsensusPathDB tool. Venn diagrams depicting the overlap of common Mbd2 binding peaks near TSSs (within 5000 bp upstream or downstream of the TSS) obtained in the present study with those in MCF-7 breast cancer cells stably transfected with MBD2 (**g**) and human mammary epithelial cells (**h**) as presented in the ChIP-Atlas dataset.

To delineate the landscape of Mbd2 binding in PyMT tumors, we performed ChIP-Seq analysis on primary breast tumors harvested from the PyMT mice at sacrifice (*n* = 3). Background subtraction was performed using the ChIP-Seq data of PyMT-*Mbd2*^*−/−*^ tumors (see Fig. 4a for the experimental workflow). A total of 3083 Mbd2 peaks were identified and annotated to 1082 unique mouse genes (*p* < 0.05) (Supplementary Table 2). For some of the genes, there was more than one Mbd2 binding peak. The Mbd2 binding peaks were dispersed across different genomic features (Fig. 4b); however, the high enrichment of peaks near transcription start sites (TSSs) indicated a strong occupancy bias for Mbd2 at gene promoters (Fig. 4c). A de novo motif discovery search revealed that the Mbd2 binding landscape was enriched with AT-rich interaction domain 5 A (Arid5a), nuclear factor of activated T cells 5 (NFAT5) and several members of the interferon regulatory factor (IRF) family (Fig. 4d). Similar enrichment of NFAT5 was observed when ChIP-Seq analysis of Mbd2 binding was performed on the mouse hippocampus^41^. When we performed analysis for known motifs, enrichment of IRF family members, NFkB-p65 and several other transcription factors was observed (Fig. 4e). We next performed pathway analyses of the annotated genes nearest the Mbd2 binding peaks and found statistically significant enrichment of several pathways related to cancer growth and progression, including p38, mitogen-activated protein kinase (MAPK), aurora A signaling, and others, as listed in Fig. 4f. We next compared the MBD2 binding peaks near TSSs ( ± 5000 bp) in a publicly available ChIP-Atlas dataset^42^ obtained from human MCF-7 breast cancer cells stably transfected with *MBD2* (NCBI GEO accession no. SRX471210) with the peaks identified near TSSs in our ChIP-Seq data from primary mouse breast tumors and found 10 overlapping genes (Fig. 4g). The poor overlap between the two datasets may be due to differences in species (human vs. mouse), the antibody used for ChIP (different sources of antibodies) and the type of sample (cell line vs. tumor tissues). When we overlapped the MBD2 binding peaks from our study with those in human mammary epithelial cells (NCBI GEO accession no. SRX758326), only one overlapping gene, amyloid beta precursor protein binding family B member 2 (*APBB2*), was identified (Fig. 4h).

### Transcriptome and proteome analyses revealed that Mbd2 modulates several important mesenchyme-associated markers during EMT

**Fig. 5:**
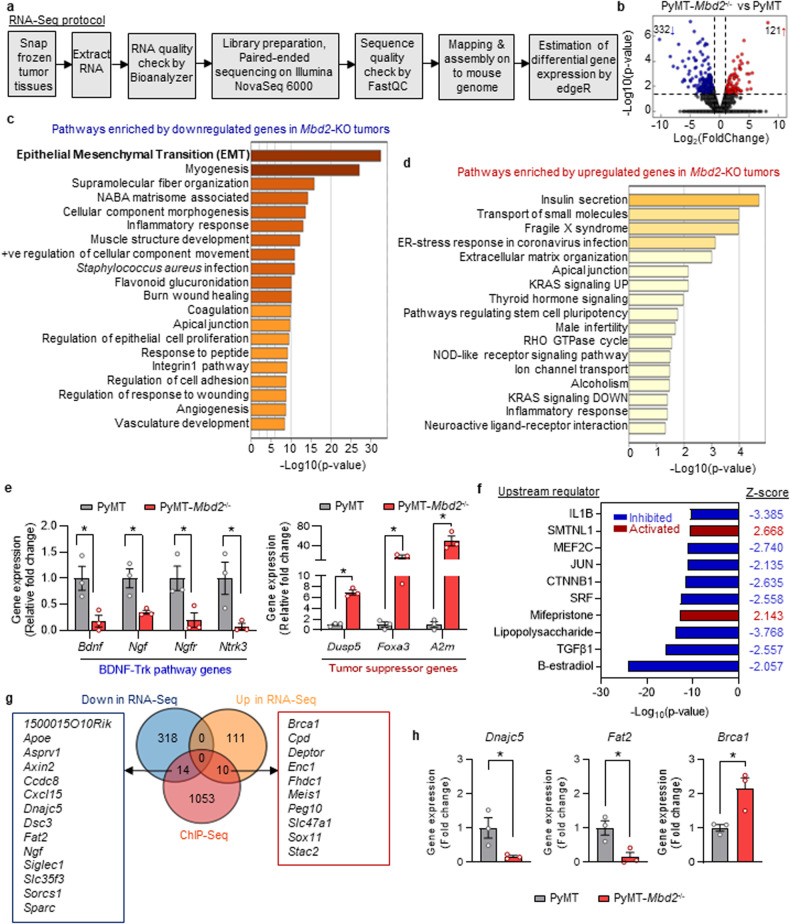
Transcriptomic and proteomic analyses of wild-type and *Mbd2*-KO PyMT tumors. RNA extracted from mammary tumors of control and *Mbd2-*KO PyMT mice was subjected to sequencing (*n* = 3 samples/group). **a** Schematic of the steps followed for RNA-Seq. **b** A volcano plot showing the DEGs (332 downregulated and 121 upregulated) in the *Mbd2*-KO vs. wild-type PyMT tumors. Pathway enrichment analysis of the significantly downregulated (**c**) and upregulated (**d**) DEGs in *Mbd2-*KO PyMT tumors. **e** qPCR validation of the selected downregulated genes related to the BDNF-Trk (*Bdnf, Ngf, Ngfr, Ntkr3*) pathway as well as selected tumor suppressor genes (*Dusp5, Foxa3, A2m*) that were upregulated in *Mbd2-*KO tumors compared to their wild-type counterparts (*n* = 3 mice/group). **f** The top ten upstream regulators of the DEGs identified via RNA-Seq, as predicted by upstream regulator analysis with the IPA application. **g** Venn diagram of genes overlapping between the RNA-Seq and ChIP-Seq data and (**h**) validation of several overlapping genes identified by the integrative analysis by qPCR (*n* = 3 mice/group). Statistical significance was determined using Student’s t-test and is shown by an asterisk.

We then investigated the transcriptomic changes triggered by genetic deletion of *Mbd2* via RNA-Seq analysis of primary tumors obtained from PyMT wild-type and PyMT-*Mbd2*^*−/−*^ mice (*n* = 3 samples/group; Fig. 5a). We found that, in comparison to those in the wild-type group, the primary tumors in the PyMT-*Mbd2*^*−/−*^ group exhibited significant differences in the expression of 453 genes ( | log2(fold change)| > 1; *P* < 0.05) (Supplementary Table 3). The expression changes occurred in both directions, but most of the differentially expressed genes were downregulated, suggesting that these genes are transcriptionally activated by Mbd2; 121 genes were upregulated and 332 genes were downregulated in the *Mbd2*-KO PyMT tumors (Fig. 5b). We performed separate pathway analyses for the differentially up- and downregulated genes using Metascape^43^ (Fig. 5c, d). We found that the top pathway enriched in the genes downregulated in *Mbd2-*KO PyMT tumors was the EMT pathway, a major driver of metastatic progression (Fig. 5c). On the other hand, the pathways enriched in the genes upregulated by Mbd2 deletion were involved in processes such as insulin secretion and the inflammatory response, as shown in Fig. 5d. Moreover, the statistical significance of the enrichment was greater for the pathways enriched in the downregulated genes than for those enriched in the upregulated genes, as shown by the negative log10(p) values on the x-axis in Fig. 5c, d. To validate the results from the RNA-Seq analysis, we performed quantitative polymerase chain reaction (qPCR) analysis of several up- and downregulated DEGs and found concordant changes in their expression in the *Mbd2*-KO PyMT tumors (Fig. 5e). We further validated the downregulation of genes encoding several key ligands [brain-derived neurotrophic factor (*Bdnf*) and nerve growth factor (*Ngf*)] and receptors [*Ngfr* (also known as p75^NTR^*)* and *Ntrk3* (also known as TrkC)] involved in neurotrophic factor-mediated Trk receptor signaling, which participates in tumor cell proliferation, survival and EMT through modulation of the PI3K, NF-κB, and MAPK pathways^44^ (Fig. 5e). Moreover, the transcriptional activation of several known tumor suppressor genes [dual specificity phosphatase 5 (*Dusp5*), forkhead box A3 (*Foxa3*), and alpha-2-macroglobulin (*A2m*)] upon *Mbd2* deletion was validated by qPCR (Fig. 5e). Dusp5 functions in dephosphorylating Erk1/2 and thereby reduces the activity of the MAPK pathway^45^, Foxa3 reduces the survival as well as the stem cell-like properties of cancer cells^46^, and A2m suppresses growth-promoting signaling pathways such as the PI3K/AKT and SMAD pathways^47^. These results partly explain the reduced activation of cancer-promoting signaling pathways in the *Mbd2*-KO PyMT tumors, as shown in Fig. 3a.

Next, we employed upstream regulator analysis (URA) with the IPA application to identify potential transcription factors, cytokines, growth factors, or chemical entities that could regulate the gene expression changes observed upon Mbd2 deletion in our transcriptome datasets (Fig. 5f). Interestingly, the top upstream regulators predicted to be downregulated included TGF-β1, serum response factor (SRF), Catenin Beta 1 (CTNNB1), and JUN, all of which are known to be involved in the EMT pathway.

Next, we performed integrative analyses of the significant DEGs identified by RNA-Seq with the 1082 mouse genes that showed enrichment for Mbd2 binding via ChIP-Seq. We found 24 overlapping genes, 14 of which were downregulated and 10 of which were upregulated upon *Mbd2* deletion (Fig. 5g). This finding suggests that most of the gene expression changes identified via RNA-Seq are due to an indirect effect of Mbd2, which changes the expression of some intermediary factors that, in turn, change the expression of downstream target genes. We then validated the expression of several overlapping genes [DnaJ heat shock protein family member C5 (*Dnajc5*), FAT Atypical Cadherin 2 (*Fat2*), and breast cancer 1 (*Brca1*)] between the RNA-Seq and ChIP-Seq data by qPCR (Fig. 5h) and found consistent changes in their expression between the RNA-Seq and qPCR data. Previous studies have shown that *Fat2*^48^ and *Dnajc5*^49^ are highly expressed in breast cancer, while *Brca1* acts as a tumor suppressor^50^. As shown by our ChIP-Seq analysis, Mbd2 binds to the regulatory sequence of *Brca1*. Once *Mbd2* gene is deleted, *Brca1* expression is upregulated, suggesting that Mbd2 represses this gene. Upregulation of *Brca1* might contribute to the tumor suppression mediated by the deletion of *Mbd2*. In contrast, Mbd2 possibly acts as an activator of *Fat2* and *Dnajc5*, as *Mbd2-*KO causes a reduction in the expression of these genes.

Emerging evidence suggests that the transcription of genes not within the protein-coding regions of the genome plays a role in the regulation of gene expression and disease pathogenesis^51^. Therefore, we next focused on examining whether lncRNAs are differentially expressed upon homozygous deletion of the *Mbd2* gene using our RNA-Seq data and found that 60 unique lncRNAs were significantly differentially expressed in *Mbd2*-KO PyMT tumors compared to their wild-type counterparts (Supplementary Table 4). Gene Ontology (GO) analysis of the lncRNAs revealed their involvement in a wide range of biological processes related to RNA processing and transcriptional regulation (Supplementary Fig. 2). Since a vast majority of the lncRNAs are still uncharacterized in the context of cancer, we focused on lncRNAs with either known biological function(s) or known human orthologs in TCGA (The Cancer Genome Atlas) breast cancer patient dataset. A curated list of 16 lncRNAs that fulfilled these criteria is shown as a heatmap in Supplementary Fig. 3a. We further validated the expression of several lncRNAs [X-inactive specific transcript (*Xist*), chaperonin containing Tcp1 and subunit 6a (*Cct6a*), and plasmacytoma variant translocation 1 (*Pvt1*)] by qPCR (Supplementary Fig. 3b), where the changes in expression were consistent with those identified by RNA-Seq. The expression of the corresponding orthologous genes in human breast cancer patients represented in the TCGA database showed that *XIST* expression was significantly downregulated, consistent with previous observations showing repression of *Xist* by MBD2^52^, while the expression of *CCT6A* and *PVT1* was significantly upregulated in patient breast tumors (Supplementary Fig. 3c). The expression of the *PVT1* gene correlated with *MYC* expression in human breast tumors according to Pearson correlation analysis of data obtained from 4307 patients (Supplementary Fig. 3d). Like their human orthologs, these two genes are also located in close proximity to each other, on chromosome 15 in the mouse genome (Supplementary Fig. 3e). A previous study also showed that *MBD2* depletion reduced *Myc* gene expression in Jurkat T cells^13^. Therefore, we checked whether *Myc* expression is differentially regulated upon *Mbd2* deletion and found significant repression of Myc expression at both the transcriptional and translational levels in PyMT-*Mbd2*^*−/−*^ tumors compared to PyMT wild-type tumors (Supplementary Fig. 3f-g). These results suggest the possible role of Mbd2 in modulating the oncogenic Pvt1-Myc axis in cancer. However, our ChIP-Seq data did not reveal enrichment of Mbd2 binding near the regulatory regions of the *Myc* oncogene, indicating that other factors regulated by Mbd2 may directly modulate *Myc* expression. One possible mechanism may involve the Wnt/β-catenin pathway, which was previously shown to be modulated by Mbd2^53^ and to play a role in the transcriptional activation of *Myc*^54^.

### Mbd2 deletion deregulates genes related to EMT

**Fig. 6:**
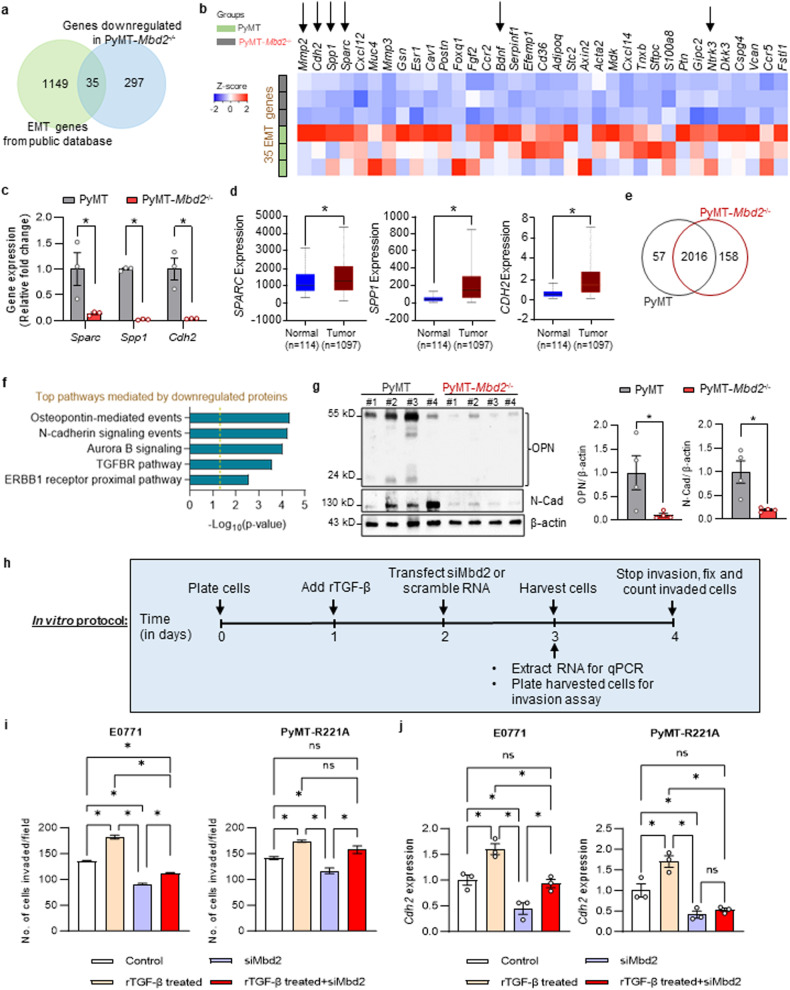
Mbd2 is a regulator of EMT in breast cancer. **a** Venn diagram showing overlap of 35 (out of the 332 downregulated) genes in *Mbd2-*KO PyMT tumors when compared with the complete repertoire of genes from the epithelial–mesenchymal transition (EMT) database. **b** The expression of the 35 EMT genes is presented as a heatmap. The genes that were validated by qPCR (in Figs. 1h, 5e, 6c) in this study are indicated by the arrows on the heatmap. **c** qPCR validation of the selected EMT-related genes (*Sparc, Spp1*, and *Cdh2*) identified by RNA-Seq was performed using tumor RNA from three mice/group. **d** The gene expression patterns of the human orthologs of the qPCR-validated genes in normal and breast tumors according to the TCGA database. **e** Venn diagram of the common and unique protein hits in wild-type and *Mbd2*-KO PyMT tumors according to the proteomics analysis. **f** Pathway enrichment analysis (PID database) of the genes significantly downregulated in *Mbd2-*KO tumors. **g** Immunoblots showing OPN and N-Cad protein expression in whole-tumor lysates (left panel). The bands at the locations corresponding to the known molecular weights of the proteins are shown. For some wild-type PyMT tumors, OPN exhibited several bands corresponding to cleaved proteins, in addition to the main band at 55 kDa. The right panel shows the densitometric quantification of the immunoblot bands plotted as the mean ± SEM values (*n* = 4 mice/group). **h** Schematic of the in vitro treatment protocol. Mouse breast cancer cell lines (E0771 and PyMT-R221A) were treated with rTGF-β and siRNA against *Mbd2*. In addition, *Mbd2* was knocked down in cells pretreated with rTGF-β. The cells in the control group were treated with scrambled RNA. **i** Next, the invasion capacity was measured by plating equal numbers of cells from the control group and the different treatment groups in a Boyden chamber with a Matrigel-coated membrane. After 18 h, the cells were fixed and stained, and cells in five randomly selected fields were counted for each sample. The results are shown as the mean ± SEM from two independent experiments with duplicate samples. **j** The expression of the mesenchymal marker gene Cdh2 in the different treatment groups was measured by qPCR. The results are shown as the mean ± SEM values (*n* = 3 samples/group). The statistically significant differences were determined using ANOVA followed by Tukey’s *post hoc* test and are shown by an asterisk. n.s.= not significant.

Since the EMT pathway was significantly enriched in the genes downregulated upon the deletion of *Mbd2* gene (Fig. 5c), we next overlapped the complete repertoire of EMT-related genes obtained from the publicly available dbEMT database^55^ with the genes downregulated in *Mbd2*-KO PyMT tumors and found 35 overlapping genes (Fig. 6a). A heatmap showing the differential expression of these 35 EMT genes in *Mbd2*-KO and wild-type PyMT tumors is shown in Fig. 6b. Of the 35 genes, 7 (*MMP2, SPARC, FOXQ1, FGF2, NTRK3, FSTL1*, and *ESR1*) were shown as having direct Mbd2 binding peaks near the TSS ( ± 5000 bp of the TSS) in the publicly available ChIP-Atlas dataset generated from human and mouse tissues^42^. To validate the results of RNA-Seq analysis, we further assessed the downregulation of several crucial EMT genes [secreted protein acidic and cysteine rich (*Sparc*), secreted phosphoprotein 1 (*Spp1*), and cadherin 2 (*Cdh2*)] by qPCR and found concordant decreases in their expression in the *Mbd2*-deleted tumors compared to that in the wild-type tumors (Fig. 6c). Notably, *Sparc, Spp1*, and *Cdh2* were found to be upregulated in the human breast cancer samples included in TCGA database (Fig. 6d), consistent with the clinical relevance of Mbd2-regulated genes in breast cancer. The *Cdh2* gene encodes N-cadherin (neural cadherin; abbreviated N-Cad) protein, a crucial mesenchymal marker that is upregulated during the ‘cadherin switch’, which ultimately leads to increased invasiveness and migratory properties in cancer cells^56^. Several studies have independently reported that Sparc and *Spp1* encoded protein osteopontin (OPN) promote cancer metastasis through the PI3K-AKT pathway^57–59^. Since we also found impairment of PI3K-AKT activation, which is required for cancer growth and invasion (Fig. 3a), we propose that Mbd2 likely regulates the PI3K-AKT pathway through transcriptional regulation of the genes encoding Sparc and OPN.

Next, we performed UHPLC/MS-MS to assess the proteomic differences between the lysates extracted from the primary tumors of wild-type and *Mbd2*-KO PyMT mice (*n* = 3 mice/group). A total of 2231 proteins were identified via our proteomics analysis. To gain insight into the biological processes affected by the deletion of the *Mbd2* gene, we focused on changes in protein abundances between the two groups as a measure of differential protein expression. This approach identified 215 proteins with differential abundances, 158 of which were upregulated and 57 of which were downregulated, in the KO samples compared to their wild-type counterparts (Fig. 6e; Supplementary Table 5). Since our transcriptome analysis revealed the downregulation of several key EMT-related genes in MBD2*-*deleted tumors, we then performed pathway analysis of the downregulated proteins in PyMT-*Mbd2*^*−/−*^ tumors; the results revealed the involvement of these genes in OPN-mediated signaling, N-cad signaling, and TGFBR pathways, all of which are involved in EMT (Fig. 6f). We next validated the downregulation of the OPN and N-cad proteins (Fig. 6g), which were also transcriptionally downregulated upon Mbd2 deletion, as shown in Fig. 6c.

We then performed integrative analysis of the differentially expressed proteins and RNAs obtained from our proteomic and transcriptomic datasets and found 10 overlapping entities, all of which showed concordant changes in their expression patterns in *Mbd2* homozygous KO tumors (Supplementary Fig. 4a, b). Interestingly, all 10 overlapping mRNAs/proteins were downregulated in the *Mbd2*-KO PyMT tumors. For example, Mbd2 deletion decreased the expression of *Gsn* (encoding Gelsolin), a protein involved in TGF-β-mediated induction of EMT in breast cancer.

Since our upstream regulator analysis with the IPA application (Fig. 5f) revealed that TGF-β1 is a potential regulator of the DEGs identified via RNA-Seq, we next asked whether *Mbd2* depletion blocks TGF-β-induced EMT. To this end, we used two mouse cell lines, E0771 and PyMT-R221A, which are of the same luminal B subtype as PyMT tumors^60,61^. We demonstrated that rTGF-β increased the invasiveness of these two cell lines in the Boyden chamber Matrigel invasion assay (Fig. 6h, i) and elevated the mRNA expression of the mesenchymal marker *Cdh2* (Fig. 6h–j). Knockdown of the *Mbd2* gene by RNA interference, which downregulated the expression of *Mbd2* in both cell lines (Supplementary Fig. 5), caused a significant decrease in cell invasion (Fig. 6i). Strikingly, when cells pretreated with rTGF-β were subjected to RNA interference by transfection of siRNA against *Mbd2*, TGF-β-induced invasion was repressed in both cell lines (Fig. 6i). We also found a concordant decrease in *Cdh2* expression in these rTGF-β-treated cells (Fig. 6j), suggesting that Mbd2 inhibition can block TGF-β-induced EMT.

Next, to rule out any species-specific idiosyncrasy, we sought to assess whether MBD2 plays a similar role in mediating EMT in human breast cancer cells. To this end, we used CRISPR interference to deplete *MBD2* in human MDA-MB-231 breast cancer cells. Depletion of MBD2 was confirmed at the protein level by western blotting (Supplementary Fig. 6a). We observed that the expression of selected genes (*SPARC*, *SPP1*, and *CDH2*) involved in EMT was indeed downregulated upon repression of *MBD2* expression (Supplementary Fig. 6); the results were consistent with those in the *Mbd2*-KO PyMT tumors shown in Fig. 6c. Since MBD2a and MBD2b are two major isoforms expressed in somatic cells^62^, we subsequently rescued the expression of the MBD2 protein by reintroducing either the full-length canonical MBD2a isoform or the MBD2b isoform, which lacks the arginine-rich domain at the N-terminus. We observed that the canonical MBD2a isoform rescued the expression of EMT genes (*SPARC*, *SPP1*, and *CDH2*) in *MBD2*-depleted cells as well as the invasive phenotype of MDA-MB-231 cells (Supplementary Fig. 6b, c). In contrast, the MBD2b isoform could not rescue the invasive phenotype even though it could rescue the expression of *SPP1* (Supplementary Fig 6b, c). Notably, another isoform of MBD2, known as MBD2c, is generated by alternative splicing and suppresses metastasis instead of inducing metastasis via MBD2a^40^. However, further studies are needed to fully understand the mechanism of gene regulation mediated by the different MBD2 isoforms.

### Mbd2 downregulates immunosuppressive Treg infiltration in PyMT tumors

**Fig. 7:**
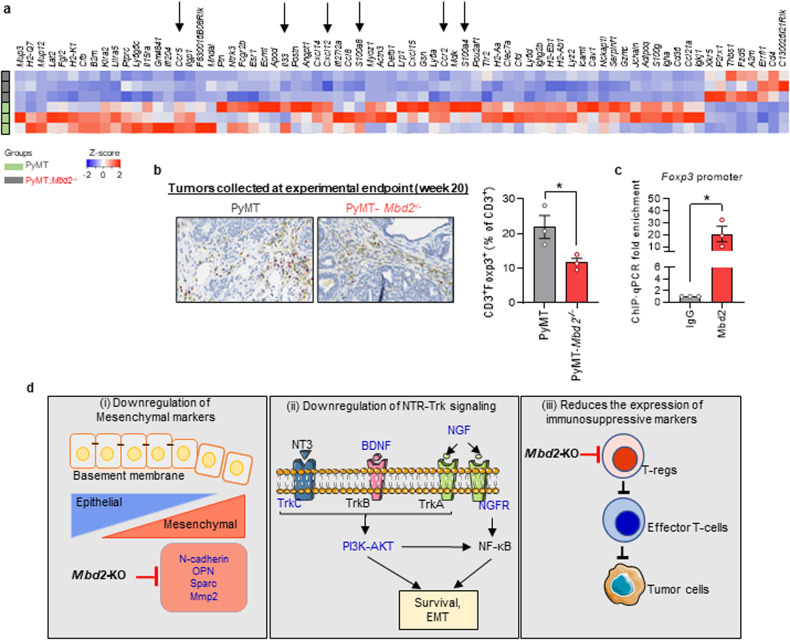
Mbd2 regulates immune-related genes in the TME. **a** Heatmap of the immune-related genes that are differentially expressed in the TME upon genetic KO of the *Mbd2* gene. The genes discussed in the text are indicated by the arrows. **b** Double staining of formalin-fixed primary breast tumor samples with antibodies against CD3 (marker for T-cells) and Foxp3 (marker for Tregs). Here, yellow staining indicates CD3, and nuclear Foxp3 staining appears purple. **c** Validation of Mbd2 binding near the promoter region of *Foxp3* by ChIP‒qPCR. Immunoprecipitation was performed using either a mouse anti-Mbd2 antibody or IgG, and the fold enrichment in Mbd2 binding was obtained via comparison with the values obtained via ChIP with IgG. The results are shown as the means ± SEMs. Statistical significance was determined using Student’s *t*-test (for the bar graphs in **b** and **c**). A *P*-value of less than 0.05 was considered to indicate statistical significance. **d** Model: (i) Mbd2 deletion downregulated key mesenchymal markers that interfere with signaling pathways related to survival and EMT. The targets with altered expression/activation in *Mbd2*-KO PyMT tumors are shown in blue font (ii). Deletion of Mbd2 likely relieves immunosuppression in the TME through either the downregulation of immunosuppressive cytokines or a decrease in the infiltration of immunosuppressive cells, such as Tregs (iii).

Dynamic crosstalk between cancer cells and immune cells within the tumor microenvironment (TME) modulates tumor evolution and progression^63^. Moreover, Mbd2 plays a crucial role in mediating the immune response^64^. Therefore, we next analyzed the RNA-Seq data obtained from breast tumors to determine whether the expression of immune-related genes is altered upon Mbd2 deletion in PyMT mice. We used the 453 DEGs as inputs to search for ‘immune’-related functions in all the pathway databases integrated within the Metascape resource^43^ and performed a manual curation of the known literature. The list of immune-related genes that were differentially expressed in the *Mbd2-*KO PyMT tumors is depicted as a heatmap in Fig. 7a; most of the genes in this list were downregulated by *Mbd2-*KO, suggesting that Mbd2 is required for the transcriptional activation of these immune-related genes. We found that several genes [C-C chemokine receptor type 2 (*Ccr2*), *Ccr5*, and C-X-C Motif Chemokine Ligand 12 (*Cxcl12*)] involved in immunosuppression during cancer progression were downregulated upon Mbd2 deletion (indicated by the arrows in the heatmap in Fig. 7a). Moreover, the expression of genes encoding various alarmin molecules, such as interleukin 33 (IL-33), S100 Calcium Binding Protein A4 (S100a4), and S100a8, which promote tumorigenesis and metastasis, was decreased in *Mbd2-*KO PyMT tumors (shown by the arrows in Fig. 7a). Therefore, we next checked whether the expression of forkhead box P3 (Foxp3), a marker for regulatory T cells (Tregs), which suppress the immune response, was altered in formalin-fixed primary tumor tissues obtained from wild-type and *Mbd2*-deleted PyMT mice. Our immunohistochemical analysis revealed that the percentage of CD3^+^Foxp3^+^ cells was significantly decreased in *Mbd2-*depleted tumors compared to wild-type PyMT tumors (Fig. 7b), which suggested that targeting Mbd2 could relieve immunosuppression within the TME. These results are consistent with a previous study showing that Mbd2 activates *Foxp3* expression^65^. We then checked whether Mbd2 binds to the regulatory region of *Foxp3* at the promoter of this gene by ChIP‒qPCR and found significant enrichment of Mbd2 at the regulatory region of *Foxp3* (Fig. 7c) in wild-type *Mbd2*-expressing mice, further suggesting that Mbd2 regulates the infiltration of immunosuppressive Tregs into PyMT tumors.

## Discussion

Previously, Mbd2 was suggested to promote tumor growth and metastasis; however, past studies were performed in cancer cell lines and xenografts in immunocompromised mice and used either antisense oligonucleotide- or siRNA-mediated knockdown approaches^16,17^. Since results obtained via these methodologies are highly confounded, the main outstanding question was whether Mbd2 plays a causal role in breast cancer. In this study, we genetically deleted *Mbd2* in the well-characterized transgenic MMTV-PyMT mouse model of metastatic breast cancer^21^. Since the gene is knocked out before conception, gene loss certainly precedes tumor initiation; thus, the study design allows conclusions to be drawn regarding the causal role of Mbd2 in breast cancer. Our data demonstrate that PyMT oncogene-driven spontaneous mammary tumor formation is delayed upon homozygous deletion of the *Mbd2* gene. Mbd2 might not be the major player in tumorigenesis, as tumor appearance is delayed but still occurs in KO mice. The late appearance of tumors in *Mbd2*^−/−^ mice might be a consequence of slowed tumor growth; therefore, tumors may reach a detectable size later in these mice. In addition to tumor progression, lung metastasis is significantly reduced upon genetic ablation of the *Mbd2* gene (Fig. 2i). This finding is consistent with that of in vitro studies in which *Mbd2* knockdown reduced cancer cell invasiveness (Fig. 6i), indicating that Mbd2 plays a multifaceted role in mammary tumor progression.

PyMT upregulates Mbd2, and upregulation of Mbd2 precedes the appearance of measurable primary tumors, consistent with the idea that Mbd2 partially mediates the effects of PyMT on cellular pathways that drive tumor progression; specifically, Mbd2 deletion inhibits the activation of these pathways. PyMT activates a series of downstream oncogenic signaling pathways (for example, the PI3K/Akt pathway) that are involved in tumor cell proliferation, tumor cell survival, inhibition of apoptosis, and promotion of metastasis^22^, and deletion of *Mbd2* gene significantly represses the activation of these pathways, as shown in Fig. 3a. Consistent with our observation, a recent study demonstrated that Mbd2 modulates the PI3K/Akt signaling cascade during fibrosis^66^. This finding is intriguing since Mbd2 is a well-established regulator of gene expression. Is it possible that Mbd2 has an additional role in signaling? One possible mechanism of Mbd2-mediated PI3K/AKT signaling impairment is downregulation of the genes encoding Sparc and OPN. As shown in the present in vivo study (Fig. 6c) and in our previous in vitro study^17^, Mbd2 regulates the gene expression of *Sparc*. The roles of Sparc and OPN in promoting cancer metastasis through the PI3K-Akt pathway are well established^57–59^. We hypothesize that the Mbd2-mediated downregulation of Sparc and OPN impairs the activation of the PI3K/Akt signaling involved in EMT. This possibility needs to be investigated in future studies. Another outstanding question is why the Mbd2 level is increased in PyMT tumors compared to the mammary fat pads of control mice. Wang et al. recently showed that TGF-β1 can increase *Mbd2* expression via the SMAD3 pathway^67^. Previous studies have shown that the TGF-β1-SMAD3 pathway plays a role in immune suppression and regulates the transcription of genes involved in metastasis^68^. It is plausible that tumor cells utilize the TGF-β1-SMAD3 pathway to increase *Mbd2* expression, which in turn drives the transcription of genes involved in metastasis.

Transcriptome analyses provide insights into the global gene expression changes mediated by *Mbd2*-KO. Although Mbd2 is generally believed to be a suppressor of gene activity through recruitment of the nucleosome remodeling and deacetylase (NuRD) complex and histone deacetylases to methylated promoters^69^ and a previous study in an isogenic model of breast cancer transformation showed that the majority of genes with differential expression following *Mbd2* knockdown were upregulated^70^, we showed here that the majority of genes affected by Mbd2 deletion were silenced rather than activated. Many of these genes play active roles in cancer growth and metastasis. Thus, Mbd2 serves as an activator of several cancer genes in our model. However, although the silencing activity of Mbd2 has been emphasized in past studies, several studies have shown that Mbd2 is also involved in gene activation. For example, Baubec et al. showed that Mbd2 plays a bimodal role in embryonic stem cells and that Mbd2 binds to methylated regions in the genome enriched with repressive marks as well as to active unmethylated regulatory regions that are enriched with DNase-hypersensitive sites and activating chromatin marks^71^. Mbd2 is most likely targeted by different factors to either activate or silence gene expression. It was proposed that the Nucleosome Remodeling and Deacetylase (NurD) complex targets Mbd2 to active unmethylated regions in embryonic stem cells^71^. Stefanska et al. showed that Mbd2 triggers hypomethylation of cancer-promoting genes in liver cancer^72^ and that the transcription factor CCAAT/enhancer-binding protein alpha (C/EBPA) recruits Mbd2 to its targets to trigger transcription initiation^73^. Mbd2 is recruited to the *Foxp3* gene in regulatory T cells, which results in loss of methylation possibly through recruitment of ten-eleven translocation 2 (Tet2)^65^.

Our RNA-Seq results indicated that several key EMT pathway-related signature genes encoding mesenchymal markers, such as N-Cad, SPARC, and OPN, were significantly downregulated by Mbd2. We previously showed that Mbd2 can directly bind to the upstream region of *SPARC* and regulate its expression^17^. For *Cdh2* and *Spp1*, we could not detect direct Mbd2 binding in our ChIP-Seq assay or identify this interaction in publicly available ChIP-Atlas datasets^42^. It is possible that MBD2 regulates and/or recruits other transcriptional activators, which in turn controls the expression of these genes. Moreover, changes in the promoter methylation status of the *Cdh2* and *Spp1* genes, as a direct or indirect effect of *Mbd2* deletion and subsequent binding by a transcriptional repressor, may also constitute a possible mechanism for regulating these genes in breast cancer. All these possibilities need to be tested in the future. EMT is a highly dynamic process that directs epithelial cells within a particular tissue to undergo multiple biochemical changes, resulting in their transformation into more-invasive mesenchymal cells^74^. Therefore, decreases in the expression of mesenchymal markers reduces the metastatic spread of primary tumors. Proteomic analysis of the lysates obtained from wild-type and *Mbd2-*KO PyMT tumors further confirmed that the top pathways enriched with the differentially downregulated proteins in *Mbd2-*KO PyMT tumors were related to OPN-, N-Cad- and TGFBR-mediated signaling events, all of which are involved in EMT (Fig. 6f). Moreover, *Mbd2* knockdown partially blocked the TGF-β-induced increase in the invasiveness of breast cancer cells in vitro. These data are consistent with emerging evidence on the involvement of Mbd2 in EMT during the progression of several types of cancer^40,75^.

In conclusion, the results of the present study indicate that Mbd2 coordinately activates and suppresses gene expression programs leading to tumor growth and metastasis, identifying it as a candidate target for breast cancer therapeutics. Specifically, we found that Mbd2 is involved in EMT during metastasis by directly modulating the expression of key marker genes or modulating molecular signaling pathways that drive EMT (Fig. 7d). Furthermore, Mbd2 helps to relieve the immune suppression mediated by Tregs. In line with these findings, deletion of *Spp1*, a target of Mbd2, has been shown to alleviate immune suppression during lung metastasis in mouse models of breast cancer^76^. A similar trend toward a reduction in immune suppression was also observed in *Spar*c KO mice^77^. Recent evidence indicates that EMT renders cancer cells resistant to antitumor immunity^78^. Therefore, it is clear that the EMT program can also be utilized to predict the response to immunotherapies. We predict that targeting Mbd2 in combination with checkpoint inhibitors would boost the antitumor immune response in vivo. However, this needs to be tested in the future. The immediate focus should be on developing effective therapeutic strategies to target Mbd2. Because of the extreme structural flexibility of the Mbd2 protein due to the presence of intrinsically disordered regions, developing drugs targeting Mbd2 is challenging^39^. To this end, several small molecule inhibitors have shown promise in preclinical settings for some cancers^39,79^. Recently, Zhang et al. utilized a liposome-based system to deliver siRNA against *Mbd2* to block cancer metastasis in vivo^75^.

Since *Mbd2-*depleted mice are viable and fertile, testing whether targeting Mbd2 and/or its ability to bind methylated DNA produces a similar anticancer effect in patients with breast cancer to reduce cancer-associated morbidity and mortality would be similarly attractive. This study opens a novel avenue for using epigenetic therapies targeting DNA methylation abnormalities in cancer cells as single-agent monotherapies or in combination with standard-of-care treatment regimens.

### Supplementary information

Supplementary figures

Supplementary Tables

## Data Availability

The authors declare that all relevant data generated as part of this study are available in the main manuscript and its supplementary files.
