# Supplementary material for: Methyl-CpG binding domain protein 2 (Mbd2) drives breast cancer progression through the modulation of epithelial-to-mesenchymal transition
Source: Exp Mol Med. 2024 Apr 1;56(4):959–74. doi: 10.1038/s12276-024-01205-2 (PMC11058268; doi:10.1038/s12276-024-01205-2)
Supplement: Supplementary file 1 — Supplementary figures [file 12276_2024_1205_MOESM1_ESM.pdf]

Supplementary Figures

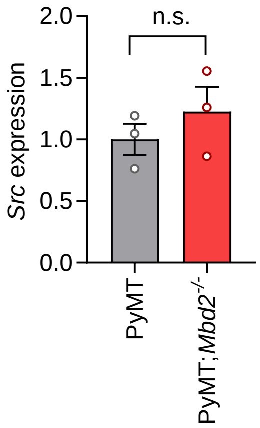

Supplementary Fig. 1: *Src* gene expression in tumors. qPCR assessing the expression of the *Src* oncogene in PyMT and PyMT-*Mbd2*$^{-/-}$ tumors (n=3 animals/group). The results are shown as mean ± SEM. No statistically significant difference was observed. n.s.= not significant.

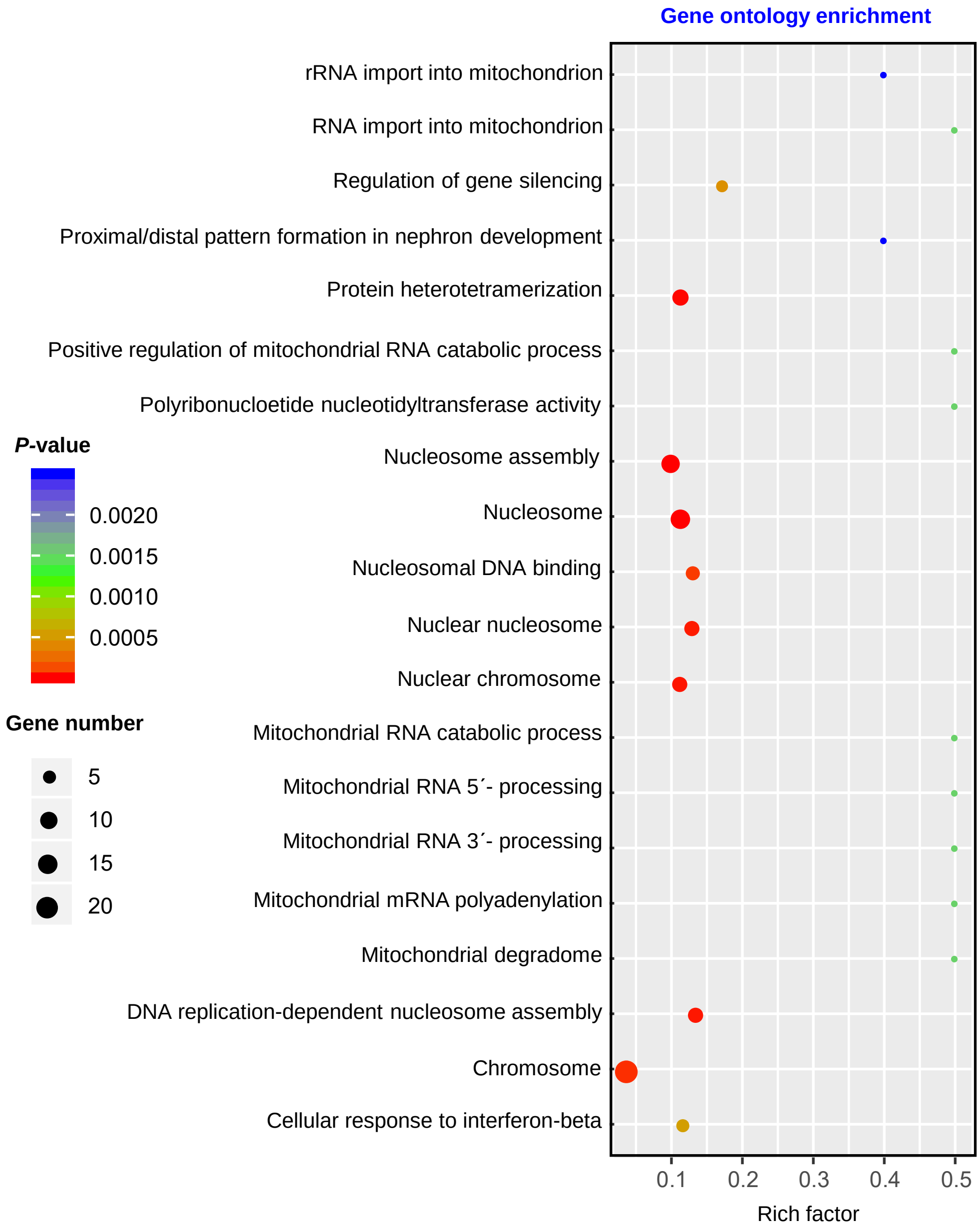

Supplementary Fig. 2: Gene ontology (GO) enrichment analyses using the list of differentially regulated lncRNAs upon deleting the *Mbd2* gene.

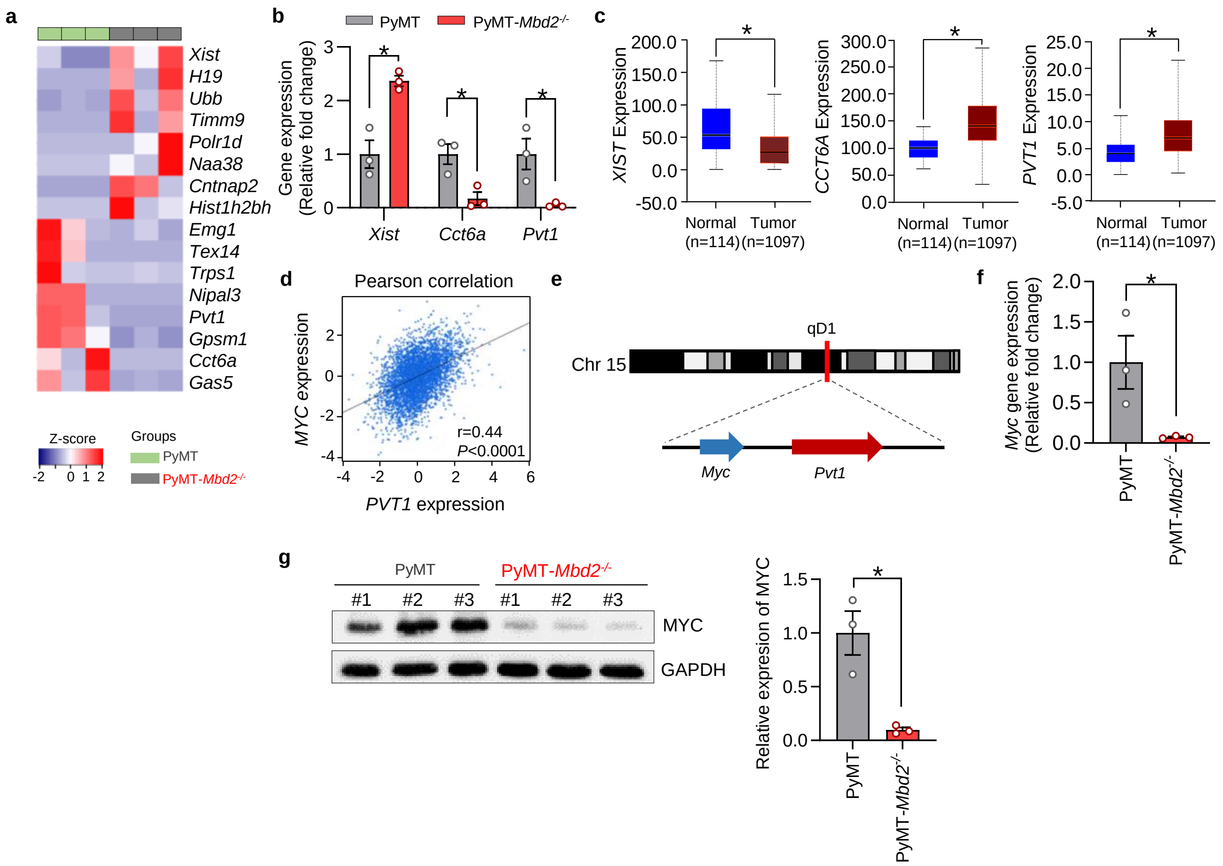

Supplementary Fig. 3: Analysis of differentially expressed lncRNAs in *Mbd2*-KO PyMT tumors. (a) Heatmap of differentially expressed lncRNAs in *Mbd2*-KO PyMT tumors with known human orthologs in the TCGA breast cancer patient dataset. **(b)** qPCR validation of the selected lncRNAs (*Xist*, *Cct6a*, *Pvt1*) obtained from RNA-Seq was done using tumoral RNA from at least three animals/group. **(c)** The gene expression pattern of the human orthologs of the qPCR validated genes in normal and breast tumors according to the TCGA database. **(d)** Pearson correlation between human *PVT1* and *MYC* genes using data obtained from 4307 patients in TCGA, GSE81538, and GSE96058. **(e)** Schematic of *Myc* and *Pvt1* genes' chromosomal location reveals the proximity of the genes on the mouse genome (not drawn to scale). **(f-g)** RNA and protein levels of Myc showed a significant decrease in *Mbd2*-KO PyMT tumors relative to wildtype controls. Results are shown as mean ± SEM (n=3 tumors/group). Statistical significance was determined using the student's *t*-test. *$P < 0.05$.

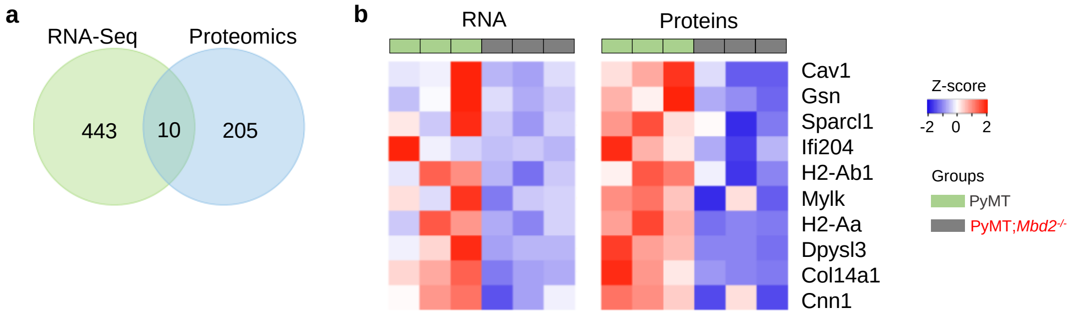

Supplementary Fig. 4: Integrative analyses of RNA-Seq and proteomics data. (a) The Venn diagram shows the overlap of 10 genes between RNA-Seq and proteomics analyses of control and *Mbd2*-KO PyMT tumors. The overlap is not statistically significant by the hypergeometric test. **(b)** A Heatmap showing the overlapped genes shows concordant downregulation in their expression on both platforms.

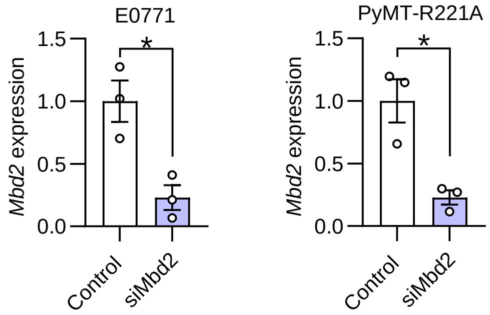

Supplementary Fig. 5: siRNA-mediated knockdown of *Mbd2*. qPCR confirms the reduced expression of the *Mbd2* gene upon transfection with Mbd2-specific siRNA (siMbd2) in mouse breast cancer cell lines E0771 and PyMT-R221A. Cells from the control groups were transfected with scramble RNA. The results are shown as mean ± SEM (n=3/group). Statistical significance was determined using the student's *t*-test. *$P < 0.05$.

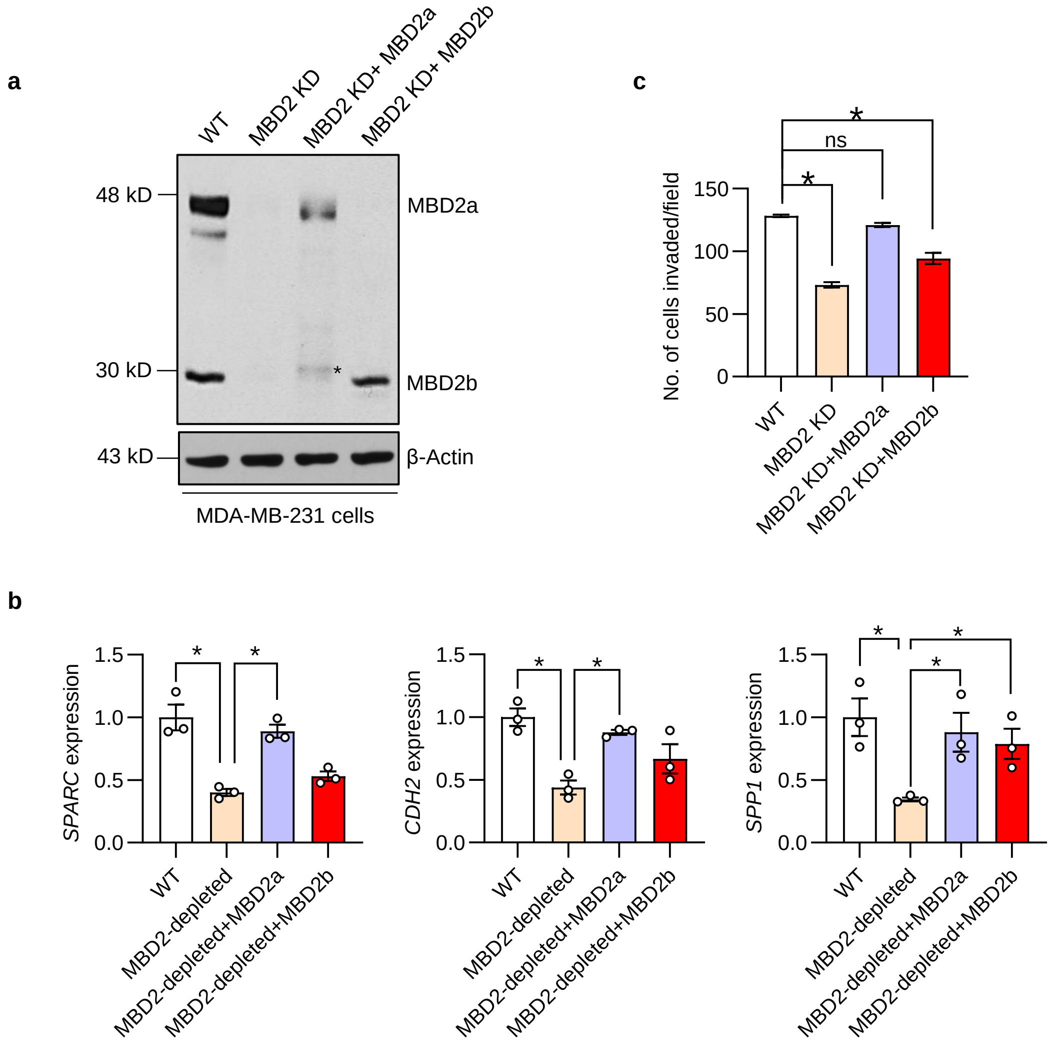

Supplementary Fig. 6: Loss-of-function followed by rescue of Mbd2 expression in human MDA-MD-231 cells. (a) Western blot confirming the CRISPR-mediated depletion of *MBD2* in MDA-MB-231 breast cancer cells. The expression Mbd2 was rescued by transient transfection with plasmids expressing the Mbd2a or Mbd2b isoforms, which showed bands of expected size. *A non-specific band that migrated slower than Mbd2b. **(b)** The expression of EMT genes (*SPARC*, *CDH2*, and *SPP1*) in different groups was assessed by qPCR (n=3/group). **(c)** The invasive capacity of the cells was measured by plating an equal number of cells from control and different treatment groups in the Boyden chamber coated with Matrigel. After 18 hours, cells were fixed and stained, and five randomly selected fields were counted and shown as bar graphs. Results are shown as the mean ± SEM from two different experiments done in duplicates. The statistically significant differences with the WT were determined using ANOVA followed by *post hoc* Tukey's test. *$P < 0.05$.
